# Primary school teachers' emotions, implicit beliefs, and self-efficacy during the COVID-19 pandemic

**DOI:** 10.3389/fspor.2022.1064072

**Published:** 2023-01-04

**Authors:** Simona Nicolosi, Martina Alba, Carmelo Pitrolo

**Affiliations:** ^1^ Faculty of Human and Social Sciences, Kore University of Enna, Enna, Italy; ^2^ School Active Kids Project, “G.G. Sinopoli” Primary School, Agira, Italy; ^3^ “Maggia” Secondary School, Stresa, Italy

**Keywords:** self-efficacy, instructional competence, online teaching, physical education, wellbeing, pandemic

## Abstract

During the Covid-19 pandemic, primary school teachers faced many challenges when providing online and hybrid teaching, especially in PE classes. This study aimed to analyze emotions, self-perceived instructional competence, and incremental beliefs during the first lockdown, in distance education instructional delivery, and, as the pandemic emergency persisted, in hybrid teaching. One hundred and four primary school teachers (Males = 7; Females = 97; M_age _= 53.24; SD_age _= 7.34) were involved in the study from four Italian Primary Schools. Participants filled in the Motivation, Emotion, Strategies, and Teaching questionnaire (MESI) ( 1) administered in an online survey. Results showed that younger teachers had significantly lower scores in negative emotions when they taught than the older ones. On the other hand, older teachers have more belief in their own abilities to improve teaching, unlike younger teachers. Multivariate regression analysis indicated that teachers' positive emotions experienced in teaching predict perceived instructional efficacy during distance learning. Furthermore, teachers' positive emotions experienced in teaching and in the role of teacher predict teachers' changes in PE teaching during distance learning. Incremental beliefs predict perceived instructional efficacy during distance learning. Efforts on pre-service and in-service teacher training programs could help teachers strengthen emotional competencies and manage their stress in the classroom, both in normal educational contexts and in adverse conditions.

## Introduction

In Italian schools, the first lockdown (from March to May 2020) led to a quick transition from face-to-face to online teaching. From the beginning of the 2020–21 school year, despite the ongoing pandemic emergency, the Italian government decided the return to face-to-face teaching for primary schools up to the first class of lower secondary school (6–11 years old), in order to guarantee pedagogical continuity and maintain the educational relationship with pupils. However, traditional teaching was often alternated with hybrid teaching, due to Covid infections forcing one or more pupils into quarantine periods. Teachers, students, and their families had to cope with a completely new situation (2, 3), implementing new strategies, changing the processes of teaching-learning, communication, and emotional relationships with students (4, 5). Primary school teachers faced many challenges when providing online and hybrid teaching, trying to quickly adapt their teaching to the contingent needs of distance or hybrid teaching. These struggles included technology issues, new strategies and approaches to planning and instruction, and new technology platforms for teachers (6).

This destabilizing situation involved the teaching curriculum as a whole, but physical education (PE) needed to be rethought due to its characteristics as a practical subject (3, 7, 8). Difficulties and barriers in teaching primary school PE existed even before the Covid-19 pandemic, in the context of traditional schooling. In Italian primary schools, PE is taught by non-specialist teachers who teach different subjects and several studies have reported on the difficulties of teachers in teaching PE (9). However, before the lockdown, many Italian primary school teachers were used to being supported by specialist PE teachers, but since the first lockdown, this support was stopped when teaching physical education online. In the context of teaching PE, self-efficacy has been analyzed as the teachers' perceived competence in being effective at implementing a new PE curriculum, adapting objectives and activities to attend to diversity in the classroom, or overcoming the most common barriers encountered in the implementation of physically active lessons (10–13). As previous studies have found, teacher efficacy is negatively associated with teacher burnout (14, 15) and positively associated with teachers' well-being (16) and instructional quality (17). Other researchers have also shown that teachers with higher efficacy develop stronger relationships with their students (18) and increase students’ engagement (19).

During the Covid-19 pandemic, the lowered self-efficacy among teachers was exacerbated. Teachers providing all-virtual instruction had the lowest levels of instructional and engagement efficacy, with respect to hybrid teachers and in-person (6). Primary school teachers showed a higher level of burnout and depersonalization in distance learning, and a lower level of efficacy in instructional strategies, especially in involving pupils with disabilities (5).

As regards implicit theories, Dweck stated that implicit beliefs refer to the individual's assumptions about basic abilities (20). Based on their beliefs about intelligence, personality and abilities, individuals construct their own personal meaning systems, structured theories which they use to think, feel and act coherently, to understand themselves and others (20). Implicit theories allow the explanation of individual differences in learning processes and outcomes, in selecting goals, and in coping with difficulties (20–23).

People with an incremental theory (growth mindset) believe that skills can be developed; they choose goals in relation to their desire to improve their skills (mastery goals). Incremental theory is associated with focusing on effort, persisting in task regardless of its difficulty and interpreting negative outcome as a learning opportunity. Incremental teachers in teaching are interested in teaching better, enhancing their skills, and facing challenges (1), they build learning contexts that orient their students towards mastery, which foster experimentation and curiosity (24).

People endorsing an entity theory (fixed mindset) believe that intelligence and ability are stable and unchangeable; they choose goals aimed to demonstrate their abilities (performance goals). Challenges are a threat to one's self-esteem and they tend to avoid situations in which they might appear to be incompetent (21, 25). Furthermore, the entity theory is associated with a negative attitude towards commitment, as in the case of experiencing failure more effort is interpreted as a lack of intelligence or ability. Consequently, an entity theory induces avoidance of challenges and low levels of persistence. Entity teachers in teaching reinforce learning situations where results and evaluations are important and assigned tasks are aimed at confirming standards in performance (1).

Both students and teachers generally tend towards one or the other theory, but it is also common to have different theories in various domains of the self and others (26).

A multitude of studies have investigated students' implicit theories, the impact of teachers in orienting the learning context towards growth or fixed mindsets, and how specific interventions can modify strong and established beliefs into incremental positions (24, 27, 28).

Furthermore, little is known about the teachers' implicit theories and how implicit beliefs influence pedagogical thinking and practice in the PE context.

Two systematic reviews on implicit theories have identified a limited number of empirical studies, most of these have involved children and adolescents in school PE (29, 30). Longitudinal studies in PE (31–33) on implicit theories in PE, have highlighted the importance for PE teachers to not only promote an incremental theory of ability but also to minimize the development of an entity theory of ability.

However, even these studies focused on the influence of teachers' implicit theories on students, but not on teachers' implicit beliefs and their meaning systems related to teaching practice.

To the best of our knowledge, although several studies have investigated the relationship between emotions and self-efficacy in primary school teachers during the Covid-19 pandemic (34, 35, 17, 5), fewer studies have analyzed emotions, incremental beliefs and self-efficacy in primary school teachers in facing PE in online teaching (3).

Thus, the purpose of the present study was to assess emotions, self-perceived instructional efficacy, and incremental beliefs during the first lockdown, in distance education instructional delivery, and, as the pandemic emergency persisted, in hybrid teaching. The hypotheses formulated are the following:

**Hypothesis 1 (H1):** Positive and negative emotions predict self-perceived instructional efficacy, during distance teaching.
**Hypothesis 2 (H2):** Positive and negative emotions predict the perceived efficacy of PE Teaching strategies, during distance teaching.
**Hypothesis 3 (H3):** Positive and negative emotions predict changes in PE teaching during distance teaching.
**Hypothesis 4 (H4):** Incremental beliefs predict self-perceived instructional efficacy, during distance teaching.
**Hypothesis 5 (H5):** Incremental beliefs predict the perceived efficacy of PE Teaching strategies, during distance teaching.
**Hypothesis 6 (H6):** Incremental beliefs predict changes in PE teaching during distance teaching.

## Materials and methods

### Procedures and sampling

A mixed-methods approach was used in this research (36). Researchers used convenience sampling to recruit teachers to complete an online questionnaire available from the end of January 2022 to the end of February 2022. This period corresponded to the second school year (2021–2022) after the return to face-to-face teaching during the pandemic. The inclusion criteria were being a primary school teacher and voluntary participation in the research.

The study was conducted after requesting the cooperation of the participating school principals and obtaining the approval of school boards. The questionnaire was distributed and filled in electronically and individually. The questionnaire was administered *via* online survey platforms (i.e., Google Forms) and accessed by participants using a designated link in their institutional email. Before answering the questionnaire, in an online consent form section, all the participants expressed their agreement and voluntary participation.

In the consent form section, the teachers were given general information about the study objectives, anonymity, data collection, and confidentiality of their responses and were allowed to refuse to participate. Then, teachers were invited to complete once a 20-min-long online questionnaire.

### Participants

Study participants were 104 Italian teachers (7 males and 97 females), aged between 32 and 65 years (M = 53.24; SD = 7.34), enrolled in 4 primary schools in 4 countryside towns in the South of Italy. The in-service teaching years of the participants ranged between 1 and 41 years (M = 23.83; SD = 10.34). Participants were then divided into two age categories by median age (32–53 years old and 54–61 years).

### Measures

An online questionnaire composed of two sections was administered. The first section contained questions on socio-demographic and professional information (i.e., gender, age, hometown, educational qualification, school and in-service years, teaching in the current year, teaching PE). In the second section, the Motivation, Emotions, Strategies and Teaching Questionnaire (MESI) by Moè, Pazzaglia, and Friso (1) was used to assess emotions, self-perceived instructional efficacy, and incremental (or entity) beliefs about teaching.

The MESI is a self-report questionnaire that consists of six subscales: job satisfaction (5 items), emotions (30 items), self-perceived instructional efficacy (24 items), teaching strategies (30 items), teaching practices (25 items), and incremental (or entity) beliefs about teaching (16 items).

In this study, three subscales were administered:

(1) *Emotions in teaching* is a 30-item subscale assessing positive and negative emotions experienced by teachers in two situations: first, as a teacher in relation to pupils and second (Emotions when I teach), as a teacher in relation to colleagues and the educational institution (Emotions in the role of teacher).
  Each item is a positive or negative emotion (e.g., “admiration”, “joy”, “shame”, “anger”). Respondents were asked to assess the frequency of each emotion experienced on a five-point Likert scale, ranging from 1 (“almost never”) to 5 (“almost always”).
  Four scores can be obtained by summing the item scores and dividing by the number of items of each subscale: Positive emotions when I teach (13 items), Negative emotions when I teach (17 items), Positive emotions in the role of teacher (13 items), Negative emotions in the role of teacher (17 items). Cronbach's alphas reported by Moè et al. (1) were respectively. 89 (Positive emotions when I teach). 89 (Negative emotions when I teach). 92 (Positive emotions in the role of teacher). 91 (Negative emotions in the role of teacher).
(2) *Self-perceived instructional efficacy:* based on Bandura's theory (10), this subscale is a 24-item subscale which measures perceived self-efficacy in a range of situations involving teaching and classroom management. Each item is a statement (e.g., “making myself understood by my students”) in which teachers were asked to indicate their rate of perceived effectiveness on a 9-point scale, from 1 = “Not at all” to 9 = “Extremely”. The score is calculated by summing the item scores and dividing by 24. (Cronbach's alpha = .96) (1).
(3) *Incremental (or entity) beliefs*: this subscale was developed on the basis of Dweck's indications (20). Each statement evaluates implicit theories about their own improving abilities in teaching. Teachers read the question header: “how much do you think each of the following skills can be improved with practice and training…” followed by 16 items representing key abilities in teaching (e.g., “motivating students”). Responses were given on a 9-point scale, from 1 = “not at all improved” to 9 = “totally improved”. The score is calculated by summing the item scores and dividing by 16. (Cronbach's alpha = .95) (1).

The internal reliability coefficients of MESI subscales for this study are shown in Table 1.

**Table 1:** Descriptive statistics and Cronbach's alphas of the subscales (overall sample *n* = 104).

MESI Subscales	Range	Cronbach's *α*	Overall			
			Min	Max	Mean	SD
Negative Emotions (in the role of teacher)	1–5	.870	1	3	1.420	.488
Positive Emotions (in the role of teacher)	1–5	.873	2	5	4.490	.511
Negative Emotions (when I teach)	1–5	.856	1	3	1.630	.388
Positive Emotions (when I teach)	1–5	.886	3	5	4.530	.499
Self-efficacy	1–9	.978	4	9	7.930	1.015
Incremental Beliefs	1–9	.988	2	9	7.730	1.522
Age			32	65	53.24	7.336
In-service years			1	41	23.83	10.344
Perceived efficacy of Teaching strategies	1–5		3	5	4.33	.689
Changes in teaching PE during on-line teaching	1–5		0	5	1.45	1.884

Participants were also asked to retrospectively provide information on Emotions, self-perceived instructional efficacy, and Incremental beliefs during the first lockdown (March-May 2020) in distance teaching and for the following two years, in alternating hybrid and face-to-face teaching.

Two further items were administered to measure the perceived effectiveness of PE teaching strategies and changes in PE teaching during distance learning. Perceived effectiveness was evaluated on a scale from 1 = “almost never” and 5 = “almost always”, perceived changes were rated on a scale of 1 to 5, 1 = “not at all” and 5 = “extremely”.

## Data analysis

All analyses were conducted with Statistical Package for the Social Sciences 28.0 (IBM Corporation, Armonk, NY, USA). The level of significance was 0.05. Means, standard deviations, and Cronbach's alpha values were performed. Correlations between all variables were conducted using Pearson's r. For the comparisons of the age groups, a one-way analysis of variance (ANOVA) on each dependent variable was calculated.

Multivariate multiple regression was used in order to explore the predictive contribution of the emotions and the incremental beliefs on the perceived effectiveness of PE teaching strategies and on changes in PE teaching during distance teaching.

## Results

The descriptive statistics and intercorrelations between all variables in the overall sample are shown in Tables 1, 2. A one-way analysis of variance (ANOVA) revealed that the younger teachers' age group (32–53 years) had significantly lower scores in negative emotions when they taught [*F* (1, 102) = 4.351 *p* < .05]. Comparisons in negative emotions as teachers in their role are close to statistical significance, also in this case with lower scores in younger teachers' age group [*F* (1, 102) = 3.610 *p* = .06]. On the other hand, teachers aged 54 to 65 believe more in their own abilities to improve teaching, unlike younger teachers [*F* (1, 102) = 4.262 *p* < .05] (Table 3).

**Table 2:** Intercorrelation between all variables (overall sample *n* = 104).

	1	2	3	4	5	6	7	8	9
1. Age	—								
2. In-service years	.758**	—							
3. Perceived efficacy of Teaching strategies	.030	−.035	—						
4. Changes in teaching PE during on-line teaching	−.153	−.089	−.115	—					
5. Negative emotions (in the role of teacher)	.226*	.241*	−.083	−.123	—				
6. Positive emotions (in the role of teacher)	−.211*	−.270**	.164	.216*	−.303**	—			
7. Negative emotions (when I teach)	.195*	.175	−.096	−.096	.864**	−.13	—		
8. Positive emotions (when I teach)	−.196*	−.222*	.198*	.131	−.243*	.923**	−.133	—	
9. Self-efficacy	.069	−.029	.281**	−.049	−.260**	.436**	−.182	.488**	—
10. Incremental beliefs	.146	.183	.116	−.135	−.134	.149	.039	.108	.408**

* *p* ≤ .01.

** *p* ≤ .001.

**Table 3:** Descriptive statistics and comparisons between age groups.

MESI Subscales	Age groups								*F*
	32–53 Years				54-65 Years				
	Min	Max	Mean	SD	Min	Max	Mean	SD	
Negative Emotions (in the role of teacher)	1	3	1.330	.428	1	3	1.510	.526	3.610
Positive Emotions (in the role of teacher)	3	5	4.560	.440	2	5	4.410	.564	2.219
Negative Emotions (when I teach)	1	2	1.550	.278	1	3	1.710	.457	4.351*
Positive Emotions (when I teach)	3	5	4.600	.433	3	5	4.460	.550	2.013
Self-efficacy	5	9	7.880	1.076	4	9	7.970	.963	.194
Incremental Beliefs	2	9	7.410	1.777	4	9	8.020	1.185	4.262*
Perceived efficacy of Teaching strategies	3	5	4.320	.741	3	5	4.330	.644	.010
Changes in teaching PE during on-line teaching	0	5	1.600	1.959	0	5	1.310	1.820	.592

* *p* ≤ .05.

Multivariate multiple regression showed that hypotheses 1 and 4 can be confirmed only partially.

Teachers' positive emotions experienced during teaching predicted perceived instructional efficacy during distance learning (RMSEA = .887 *R*^2 ^= .27 *p* < .001; Positive emotions when I teach: *β *= 1.316 *t* = 2.79 *p* = .006) (Table 4). Teachers' positive emotions experienced in teaching and in the role of teacher influenced teachers' changes in PE teaching during distance learning, only nearly to the statistically significant level of .05 (RMSEA = 1.837 *R*^2 ^= .09 *p* = .06; Positive emotions when I teach: *β *= 1.904 *t* = 1.95 *p* = .05; Positive emotions in the role of teacher: *β* = 2.555 *t* = 2.55 *p* = .01).

**Table 4:** Multivariate regression analyses testing the influence of emotions on self-perceived instructional efficacy (*n* = 104).

Independent variables	Coefficient	SE	*t*	Lower 95%	Upper 95%
Negative emotion when I teach	−.518	.400	−1.29	−1.311	.275
Positive emotions when I teach	−.447	.483	−0.93	−1.404	.511
Negative emotion in the role of teacher	.234	.481	0.49	−.720	1.189
Positive emotions in the role of teacher	1.316	.472	2.79**	.378	2.254
Constant	3.359	.946	4.79***	2.451	6.205

Dependent variable: Self-perceived instructional efficacy (RMSE: .887; *R*^2 ^= .27; *F* = 8.989***).

* *p* ≤ .05.

** *p* ≤ .01.

*** *p* ≤ .001.

Incremental beliefs predicted perceived instructional efficacy during distance learning (RMSEA = .931 *R*^2 ^= .17 *p* < .001; *β *= .2721 *t* = 4.51 *p* < .001) (Table 5). Hypotheses 2, 3, 5, and 6 were not confirmed.

**Table 5:** Multivariate regression analyses testing the influence of incremental beliefs on self-perceived instructional efficacy, perceived efficacy of delivering online PE teaching, and changes in PE during distance learning (*n* = 104).

Dependent variables	RMSE	R^2^	F	Coefficient	SE	*t*	Lower 95%	Upper 95%
Self-perceived instructional efficacy	.931	.17	20.366***	.272	.060	4.51***	.152	.392
Perceived efficacy online PE teaching	.687	.013	1.395	.052	.044	1.18	−.036	.141
Changes in PE during distance	1.876	.018	1.901	−.167	.121	−1.38	−.408	.073

Independent variable: Incremental beliefs.

* *p* ≤ .05.

** *p* ≤ .01.

*** *p* ≤ .001.

## Discussion

This study aimed to analyze emotions, self-perceived instructional efficacy, and incremental beliefs during the first lockdown, in distance education instructional delivery, and, as the pandemic emergency persisted, in hybrid teaching in a sample of Italian primary school teachers.

During the lockdown period, younger teachers had significantly lower scores in negative emotions when they taught than the older ones. On the other hand, older teachers had more belief in their own abilities to improve teaching, unlike younger teachers. These results confirm the evidence reported in a previous study (37) which demonstrated the presence of high levels of emotional exhaustion in older teachers with years of experience. Younger teachers, instead, expressed enthusiasm and performed their professional roles with serenity, without externalizing any emotional stress (37).

As Varea et al. (38) noted, interacting through different technologies led to a change in PE pedagogies, but central aspects of the educational relationship got lost in the shift to online teaching. The pandemic, in excluding social contexts and limiting physical contact, produced a shift in spaces, times and roles of teaching. This comprehensibly resulted in negative feelings, uncertainty and unfamiliar pedagogies, especially for older teachers. In spite of this, it would seem older and more experienced teachers assumed a more mastery-oriented meaning system containing adaptability, flexibility in the face of difficulties, and more coping strategies.

Managing stress levels and emotional skills could help teachers to be able to use theirs appropriately in their relationships with pupils and colleagues. This capability includes the understanding of emotions, the ability to identify, accept, evaluate, and express emotions, and the improvement of effectiveness in teaching work. In order to better manage stressful situations (17, 37).

Also, Pellerone (5) found similar findings in burnout levels, instructional control, socio-emotional skills, and teaching strategies.

Results confirmed that the positive emotions experienced in teaching predict perceived instructional efficacy during distance learning. Furthermore, positive emotions experienced in teaching and in the role of teacher predict teachers' changes in PE classes during distance learning. Incremental beliefs predict perceived instructional efficacy during distance learning.

Positive emotions are protective factors for teachers in coping with difficulties. Positive emotions experienced with pupils affected instructional efficacy while positive emotions experienced with colleagues and in the scholastic organization affected confidence in the possibilities of changing/improving PE teaching. In addition, personal beliefs related to engagement in professional self-improvement produced positive effects on instructional efficacy during distance learning.

The present research findings demonstrate that teachers' emotions, self-efficacy, and incremental beliefs are relevant in managing teaching challenges during the pandemic crisis and also in normal circumstances. As Pozo-Rico et al. (17) showed, training on emotional competencies is key to teaching because managing stress in the classroom is linked to teachers' well-being and supporting school policies.

*Future recommendation.* Teacher training programs could help teachers manage their stress in the classroom, both in normal educational contexts and in adverse conditions.

Moè observed that negative emotions should be compensated by positive ones, which can mitigate the former (1). Then with a view to fostering teachers' well-being, it would be appropriate to focus on increasing positive emotions, rather than curbing negative emotions. Below-average negative emotions, on the other hand, could indicate a lack of involvement, an underestimation of problems or a lack of a critical sense (1). Therefore, from a practical perspective, a psychological intervention addressed to teachers should consider the personal meanings assigned to each emotion – positive and negative – but also the relationship between positive and negative emotions within various professional situations. Furthermore, a group sharing of emotional experiences in the work environment could trigger a positive exchange between younger and older teachers.

In another study, we noted that during the pandemic, despite approximately half of the participants having adapted their teaching strategies to cope with the period of distance learning, the previous difficulties related to the teaching of PE probably caused 87.5% of the teachers to cease their PE lessons. Also, other recommendations concern the enhancement of instructional delivery in online PE teaching (39).

In their study on the implementation of online teaching activities in physical education classrooms, Yu and Lee (40) indicated that teachers need more time to create active learning strategies, design and implement teaching materials, including videos in distance learning. It is crucial that teachers know the web environments before teaching online, in order to focus on interactions with students and various aspects of the teaching-learning process.

Therefore, efforts in pre-service and in-service teacher training programs should focus on strengthening of cooperative networking teaching methods, PE online teaching methods as well as the use of learning distance technologies.

This study has some limitations. Firstly, the self-reported measures and the retrospective assessment of the variables before the lockdown could have created recall biases. Secondly, our study regards a non-probabilistic sampling of Italian primary school teachers, and therefore, generalizing results is potentially critical. However, we can assume that other educational settings faced similar challenges during the COVID-19 pandemic, with teachers adapting to online teaching during complete or partial school closures.

## Data Availability

The raw data supporting the conclusions of this article will be made available by the authors, without undue reservation.
